# A Radionuclide Generator of High-Purity Bi-213 for Instant Labeling

**DOI:** 10.3390/pharmaceutics13060914

**Published:** 2021-06-21

**Authors:** Stanislav Ermolaev, Aino Skasyrskaya, Aleksandr Vasiliev

**Affiliations:** 1 Institute for Nuclear Research of Russian Academy of Sciences, 60-Letiya Oktyabrya Prospekt 7a, 117312 Moscow-Troitsk, Russia; aks@inr.ru (A.S.); vasiliev@inr.ru (A.V.); 2 Department of Chemistry, Lomonosov Moscow State University, GSP-1, Leninskie Gory, 119991 Moscow, Russia

**Keywords:** targeted alpha therapy, Bi-213, ^225^Ac/^213^Bi generator, AG MP-50, extraction chromatography, Actinide Resin, DTPA, DOTA

## Abstract

A new two-column ^225^Ac/^213^Bi generator was developed specifically for using ^225^Ac containing an impurity of long lived ^227^Ac. The parent ^225^Ac was retained on the first Actinide Resin column, while ^213^Bi was accumulated on the second column filled with AG MP-50 resin via continuous elution and decay of intermediate ^221^Fr. The ^213^Bi accumulation was realized in circulation mode which allowed a compact generator design. It was demonstrated that ^213^Bi could be quickly and effectively extracted from AG MP-50 in form of complexes with various chelating agents including DTPA and DOTA. The performance of the generator presented and a conventional single-column generator on the base of AG MP-50 was tested and both generators were loaded with ^225^Ac containing ^227^Ac impurity. The ^213^Bi generation efficiencies were comparable and greater than 70%, whereas the developed generator provided a deeper degree of purification of ^213^Bi from Ac isotopes and decay products of ^227^Ac.

## 1. Introduction

The radionuclide, ^213^Bi, is considered as one of the promising radionuclides for targeted therapy of cancer [1,2]. Radiopharmaceuticals containing ^213^Bi are successfully passing clinical trials for the treatment of leukemia [3], NHL [4], carcinoma [5], neuroendocrine tumor [6], glioma [7], and melanoma [8]. In many cases, radiopharmaceuticals ^213^Bi-DOTATOC and ^213^Bi-PSMA-617 have shown high efficacy even after treatment with conventional therapies has failed [6,9].

The radionuclide, ^213^Bi, is a decay product of another promising alpha emitter ^225^Ac, which is used by itself [10,11] or as a parent radionuclide for ^225^Ac/^213^Bi generators. A single-column “direct” generator developed at the Institute of Transuranic Elements (ITU) and based on a strongly acidic cation-exchange sorbent AG MP-50 [12] is most commonly used. Other generators based on extraction chromatographic [13,14], ion exchange [15], and inorganic sorbents [16,17] are also available.

The radionuclide, ^213^Bi, has a relatively short half-life (46 min) and, therefore, it is preferable to use low molecular weight peptides and antibodies with fast pharmacokinetics as vectors for its delivery. For the same reason, significantly high activities of ^213^Bi are required to achieve a therapeutic effect. Depending on the vector molecules, a possible range of ^213^Bi administered dose may vary within 5–10 GBq [1].

Carrier-free ^225^Ac is applied as a source of ^213^Bi for conducting clinical trials of ^213^Bi-containing radiopharmaceuticals. It is obtained by the generator method from ^229^Th (T_1/2_ = 7.340 y) and the stock supplies are extremely limited. The world production of ^225^Ac by this method is no more than 70 GBq per year [2]. The main alternative method of ^225^Ac production is the irradiation of natural thorium with medium-energy protons. This method is being actively developed at INR RAS (Moscow-Troitsk, Russia) [18,19,20,21], TRIUMPH (Canada) [22], and LANL-BNL-ORNL (tri-lab, USA) [23]. The routine production of actinium will be launched at the accelerators of these scientific centers in the coming years, which makes it possible to produce several curies of ^225^Ac in one irradiation session (7–10 days). ^225^Ac from irradiated thorium contains a chemically inseparable impurity of long-lived ^227^Ac (~0.2% at the end of bombardment, EOB) [19]. Furthermore, although it was shown that such actinium can be used for therapy along with those that are generator-produced without any side effects [24], this imposes additional requirements on the ^225^Ac/^213^Bi generator. The generator system should not only provide a high yield of ^213^Bi in its rapid production and possess high radiation resistance [25] but also ensure a high degree of purification from both actinium isotopes and ^227^Ac decay products (^227^Th and ^223^Ra).

The article presents and investigates an original model of ^225^Ac/^213^Bi generator intended for utilizing the parent ^225^Ac with ^227^Ac impurity. The generator provides a fast production of ^213^Bi deeply purified from actinium isotopes ^227^Th and ^223^Ra. It consists of two chromatographic columns; ^225^Ac is firmly fixed on the first column and, as a result of mobile phase circulation, ^213^Bi is separated from ^225^Ac via continuous elution and decay of intermediate ^221^Fr and is concentrated on the second column. ^213^Bi can be efficiently and quickly stripped off by complexing agents (DTPA, DOTA, etc.), which greatly simplifies the labeling procedure.

## 2. Materials and Methods

All chemicals were of p.a. (pro analysis) quality or higher, obtained from Merck (Darmstadt, Germany), and used without additional purifications. All experiments were carried out using de-ionized “Milli-Q” water (18 MΩ·cm^−1^). Actinide Resin (P,P′-di(2-ethylhexyl)methanediphosphonic acid as an extracting agent on a silica support) with 100–150 μm particle size was obtained from Triskem, France. Biotechnology grade AG MP-50 (strong-acid cation exchange resin composed of sulfonic acid functional groups attached to a styrene divinylbenzene copolymer lattice) with 106–250 μm particle size was obtained from BioRad, Hercules, CA, USA.

γ-Ray spectroscopy with high-purity Ge-detector (HPGe) GR3818 (Canberra Industries, Inc., Meriden, CT, USA) was used for radionuclide determination. γ-Ray spectra were analyzed using software Genie 2000 (Canberra Industries, Inc., Meriden, CT, USA) and BNL-database [26].

The experiments were carried out at the temperature of 21 ± 2 °С.

### 2.1. Radionuclides

^225^Ac and ^223^Ra were produced at the linear proton accelerator of the Institute for Nuclear Research of the Russian Academy of Sciences (INR RAS, Troitsk-Moscow, Russia) by irradiation of thorium plates (1–2 mm thick) with protons of initial energy 120 MeV and isolated by liquid–liquid extraction, ion-exchange, and extraction chromatography in accordance with the reported procedures [20,27]. Impurity of ^227^Ac in actinium was determined as ~0.2% of ^225^Ac activity calculated at EOB. The yield and purity of the obtained radionuclides were controlled by γ-ray spectroscopy. The γ-lines 218.1 keV (abundance 11.4%), 440.5 keV (abundance 25.9%), 236.0 keV (abundance 12.9%), and 154.2 keV (abundance 5.7%) were chosen for ^221^Fr (T_1/2_ = 4.9 min), ^225^Ac (T_1/2_ = 9.9 d, through ^221^Fr), ^213^Bi (T_1/2_ = 46 min), ^227^Th (T_1/2_ = 18.7 d), and ^223^Ra (T_1/2_ = 11.43 min) quantification, respectively.

The stock solution of ^207^Bi (T_1/2_ = 31.55 y) in 1 M HCl (0.1 MBq/mL) was purchased from JSC Cyclotron (Russia). The γ-line 569.7 keV (abundance 97.7%) was used for ^207^Bi determination.

### 2.2. Batch Adsorption of Ra(II) and Bi(III) onto AG MP-50 Resin

Since AG MP-50 could contain impurities of calcium [28], it was previously purified. The resin was packed into large columns (1 cm inner diameter) and the calcium was eluted with 5 M HNO_3_, until the eluate was free from calcium, rinsed with distilled water, and dried at 60 °C to constant weight.

The adsorption of ^207^Bi and ^223^Ra from nitric and hydrochloric acid solutions onto AG MP-50 was investigated by mixing 5 mL of the acid solution with 0.1 mL of ^207^Bi or ^223^Ra containing spike and 0.05 g of the sorbent in plastic tubes. The samples were prepared with various acid concentrations (0.05–1 M), taking into account the content of nitric or hydrochloric acid in the spike solution. Tubes were shaken at room temperature for 40 min. According to the preliminary kinetic studies, this time period was enough to reach the sorption equilibrium. The amount of 1.5 mL aliquots of the aqueous solutions was taken for γ-ray spectroscopy after equilibration. Mass distribution ratios (D_m_), mL/g, were calculated using the well-known equation [29].

Measured activities of ^223^Ra were corrected for decay. Uncertainties of activity measurements did not exceed 10%.

### 2.3. Column Measurement of Capacity Factors of Fr(I) onto Actinide Resin

^221^Fr was separated from ^225^Ac adsorbed onto the Actinide Resin column (0.3 or 0.4 mL) by passing neutral (pH ~6) solutions of NaCl or NH_4_Cl in a concentration range of 0.0017–0.1 M. The flow rate controlled by a peristaltic pump varied from 0.2 to 1 mL/min. The eluate was collected by portions during 10–20 min. In each portion, the ^221^Fr activity was measured immediately and decay-corrected to the end of portion sampling. Capacity factors (*k’*) were estimated according to the procedure reported [30].

### 2.4. Column Measurement of Capacity Factors of Fr(I) and Bi(III) onto AG MP-50 Resin

Four columns were filled with AG MP-50 resin of volume 0.1 mL each. The columns were connected in series and attached to the exit of ^225^Ac-loaded Actinide Resin column. Solution of 0.25 M HNO_3_ or 0.017 M NaCl was passed through the column assembly for no less than 4 h. The flow rate controlled by a peristaltic pump varied from 0.3 to 2.8 mL/min. Having finished the elution, the columns were dismounted and the ^221^Fr and ^213^Bi activities in each column were measured and decay-corrected to the end of elution. Capacity factors (*k’*) were estimated according to the procedure reported [30].

### 2.5. Preparation and Milking ^225^Ac/^213^Bi Generator “Afrabis”

**Figure 1:**
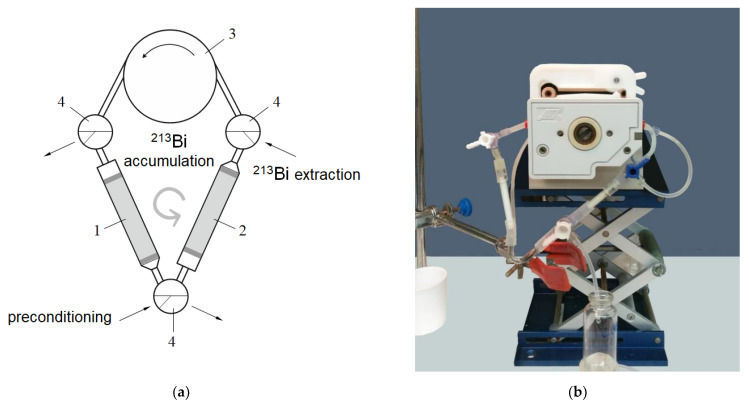
Scheme (**a**) and photo (**b**) of a laboratory ^225^Ас/^213^Bi generator “Afrabis”. 1—chromatographic column with parent ^225^Ac; 2—chromatographic column for ^213^Bi accumulation; 3—peristaltic pump; 4—three-way cock.

The amount of 0.1 MBq of ^225^Ac (with the ^227^Ac impurity) was dissolved in 5 mL of 0.1 M HNO_3_ and loaded with the help of a peristaltic pump slowly on the pre-packed and conditioned column containing 0.4 mL of Actinide Resin. The second column was filled with 0.4 mL of AG MP-50 resin and both columns were connected to form a closed-loop circuit as shown in Figure 1. The Actinide Resin column was scanned on a γ-ray spectrometer through a 1.5 mm wide slit between the lead blocks immediately after preparation and after milking.

One cycle of a possible procedure for ^213^Bi generation was as follows:

1. The first column was washed from the accumulated ^223^Ra with 15–20 mL of 0.25 M HNO_3_;
2. Then, continuous separation of the intermediate daughter radionuclide ^221^Fr from ^225^Ac for ~4 h was occurring circularly (Figure 1) at flow rate 1–1.5 mL/min;
3. ^213^Bi was desorbed from the AG MP-50 column with six 0.5 mL portions of a 0.1 M HCl/0.1 M KI solution at flow rate 1 mL/min.

The desorption of ^213^Bi from an AG MP-50 column with DTPA solutions of various concentrations and pH values was studied. An acetate buffer solution was prepared: 3.33 g of sodium hydroxide was mixed with 5.7 mL of glacial acetic acid and the total volume was adjusted to 100 mL with distilled water (pH 5.3). DTPA was dissolved in the resulting solution to the desired concentration (10^−2^–10^−5^ M). A solution with the desired pH value (2–5.3) was obtained by adding solutions of sodium hydroxide and nitric acid. The amount of 5 mL of 0.25 M HNO_3_ containing ^207^Bi spike was slowly loaded with the help of a peristaltic pump on the pre-packed and conditioned column containing 0.4 mL of AG MP-50. Bi was eluted from the column with 5 mL of DTPA solution in an acetate buffer with DTPA concentration and pH value in the investigated range. After that, the column was washed with 5 mL 6 M HCl for desorption of residue Bi and pre-conditioned with 0.25 M HNO_3_ for the next Bi sorption.

In order to test desorption of ^207^Bi with a DOTA solution, the AG MP-50 resin with ^207^Bi adsorbed on it was removed from the column, mixed with 3 mL of 10^−5^ M DOTA, and placed in a thermostat at 90 °C with stirring for 5 or 10 min. The solution was decanted through a quartz wool filter and measured by γ-ray spectroscopy.

### 2.6. Preparation and Milking A Single-Column ^225^Ac/^213^Bi Generator with AG MP-50

The amount of 0.1 MBq of ^225^Ac (with the ^227^Ac impurity) was dissolved in 2.8 mL of 4 M HNO_3_ and loaded slowly with the help of a peristaltic pump on a pre-packed and conditioned column filled with 0.33 mL of AG MP-50 resin. Afterwards, the column was washed with 1 mL 0.05 M HNO_3_, 2 mL 2 M HCl, and finally with 0.01 M HCl [12,31]. As a result, ^225^Ac was distributed within the first half of the column. The column was scanned on a γ-ray spectroscopy through a 1.5 mm wide slit between the lead blocks. 

The ^213^Bi was eluted with a mixture of 0.1 M NaJ/HCl solution followed by washing with 0.01 M HCl. The resulting ^213^Bi solution with a total volume of 3 mL was measured on a γ-ray spectrometer immediately after milking and again 6 h later. The latter measurement was conducted after the complete decay of ^213^Bi to determine the impurity of long-lived radionuclides—^225^Ac, ^227^Th, and ^223^Ra.The generator was stored wet in the 0.01 M HCl until the next use. Seven elutions of ^213^Bi were carried out during 1 month of the generator exploitation.

## 3. Results and Discussion

The ever-growing application of radionuclide generators in nuclear medicine [32] stems from the fact that a short-lived daughter radionuclide can be repeatedly reproduced and used for a long period of time; this is mainly dependent on the half-life of the parent radionuclide. Operation of most generators comprises two basic stages: accumulation and separation. First a daughter radionuclide accumulates up to transient equilibrium with the parent. Usually, both radionuclides reside together in a generator at this stage. Then the daughter is separated and recovered from the generator as fast as possible to reduce decay losses and the next generation cycle begins.

Operation mode of the generator presented here and named “Afrabis” (acronym: A—actinium-225, fra—francium-221, and bis—bismuth-213) is different. Formation and concentration of ^213^Bi take place in the course of permanent separation of intermediate ^221^Fr from ^225^Ac fixed on a mother column (Figure 1) and its decay.

As a result of dynamic accumulation arranged in circulation mode, ^213^Bi proves to already be removed from the mother column and concentrated on the second column to the moment when it reaches the transient equilibrium with ^225^Ac (it takes ~5–6 half-lives of ^213^Bi). Finally, ^213^Bi is extracted in a small volume of appropriate solution. Overall, ^213^Bi yield consists of the efficiency of single steps, namely, ^221^Fr elution, ^213^Bi concentration, and subsequent extraction from the generator.

### 3.1. Elution of ^221^Fr from the Column Containing ^225^Ac

**Figure 2:**
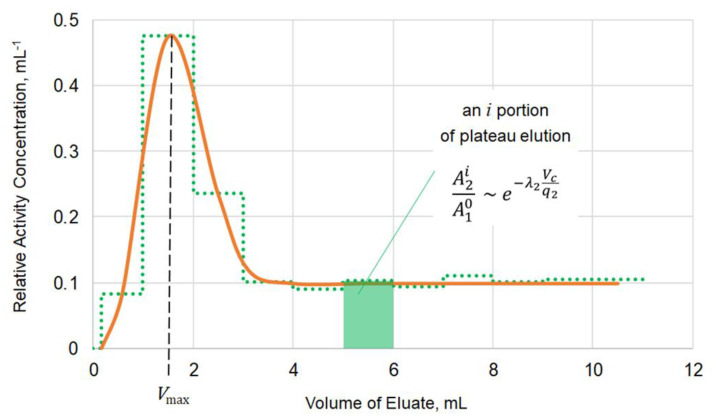
Differential (mL^−1^) elution curve of ^221^Fr eluted from the column containing 0.3 mL Actinide Resin with 0.0025 M NH_4_Cl solution passing at flow rate of 1.0 ± 0.1 mL/min. A dotted histogram and a solid curve represent experimental data and a fitting line, respectively. Designations: $A_{2}^{i}$—^221^Fr activity in an *i* portion of plateau elution corrected for decay to the end of portion sampling; $A_{1}^{0}$ —^225^Ac activity at the start of elution; Vcq2 —residence time of ^221^Fr in the chromatographic column (see explanation in the text below).

Strong retention of ^225^Ac is necessary for long-term elution of ^221^Fr; this is why the extraction chromatographic sorbent Actinide Resin demonstrating high affinity to Ac(III) in mineral acid media [33,34] was chosen for a mother column. The behavior of ^221^Fr on Actinide Resin was studied via relatively fast elution by portions and the activity of ^221^Fr in each portion is corrected towards the end of portion sampling. The typical shape of ^221^Fr elution curve is shown in Figure 2.

The first part of the curve represents, mainly, the elution of ^221^Fr, which was in transient equilibrium with ^225^Ac at the start of elution. This part of ^221^Fr was stripped off in a small bolus-like volume of eluate. The volume *V_max_* corresponding to maximum of bolus peak (Figure 2) was used for evaluation of capacity factor *k’* of Fr(I):

(1) k ’=Vmax−VcVc=1−q2Qq2Q

where *V_c_* denotes the free volume of sorbent packing accessible for mobile phase (eluent), *Q* denotes flow rate of eluent, and *q_2_* denotes the rate of ^221^Fr movement through the sorbent.

Then, the elution curve reached a plateau caused by the amount of ^221^Fr produced during the elution and immediately washed out. It is the ^221^Fr that is responsible for ^213^Bi production in the generator “Afrabis”. The activity of ^221^Fr in a portion of plateau-part of the elution curve depends on the residence time *V_c_/q_2_* of ^221^Fr in the chromatographic column [30]. The values of *q_2_* obtained from the plateau-part of curve also served for k’ Fr(I) evaluation. Normally, the k’ values determined from both bolus and plateau parts of elution curve were close.

**Figure 3:**
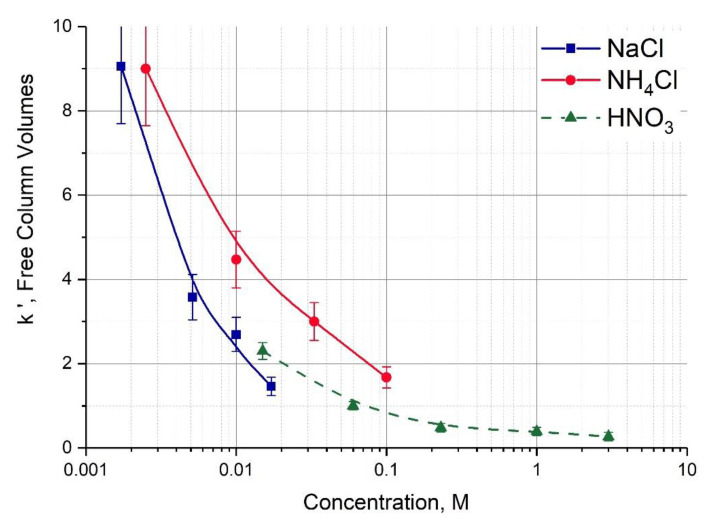
The capacity factor k’ of Fr(I) upon sorption onto Actinide Resin as a function of NaCl and NH_4_Cl concentrations. The literature data [30] for HNO_3_ solutions are shown by dotted line.

^221^Fr was easily separated from ^225^Ac adsorbed onto Actinide Resin with diluted solutions of mineral acids [30]. Elution of ^221^Fr from a mother column filled with inorganic sorbent on the base of TiO_2_·xH_2_O was carried out with neutral NH_4_Cl solutions [35]. In this work, we report data on capacity factor k’ of Fr(I) onto Actinide Resin in neutral (pH ~6) NaCl and NH_4_Cl solutions (Figure 3).

The efficiency of ^221^Fr elution with HNO_3_ and salt solutions was found to be comparable. This means that we are able to use neutral diluted NaCl solution, e.g., physiological saline, in the generator “Afrabis” at the stage of ^213^Bi accumulation, which reduces radiolytic impact on the resin.

**Figure 4:**
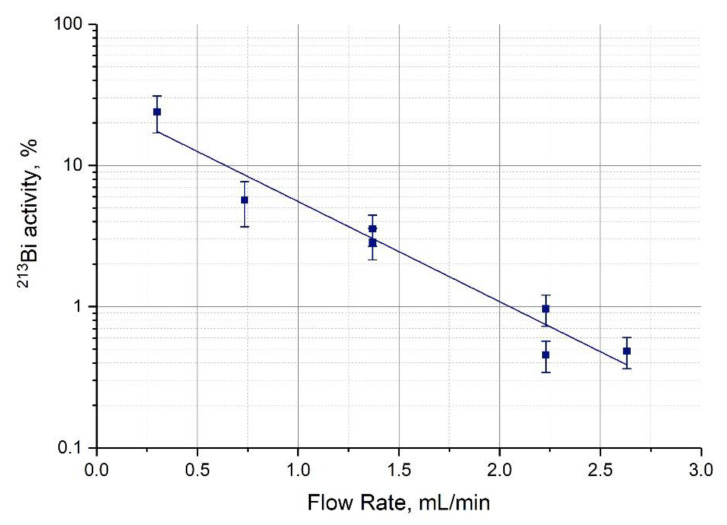
^213^Bi residue on the column containing 0.4 mL Actinide Resin (% to equilibrium activity) depending on the flow rate of mobile phase (0.25 М HNO_3_) during the accumulation stage.

Depending on the time of residence in column, some part of ^221^Fr decays into ^213^Bi, for which its retention on Actinide Resin is rather strong. For example, the k’ value of Bi(III) in 0.25 M HNO_3_ was estimated at 4·10^3^ [30]. Therefore, it seems reasonable to use the flow rate of mobile phase as high as possible in order to diminish ^213^Bi losses on the mother column during the accumulation stage, as it is shown in Figure 4.

However, the increase of flow rate results in the growth of solution volume passed through the mother column because the duration of the accumulation stage cannot be significantly shortened and is determined by the time required for attaining the transient equilibrium of ^213^Bi to ^225^Ac (~5–6 half-lives of ^213^Bi). The large solution volume may, in turn, promote the breakthrough of ^225^Ac and ^227^Ac/^227^Th adsorbed on Actinide Resin (data on the distribution of long-lived radionuclides along the Actinide Resin column is reported below). Thus, the choice of optimal flow rate for the accumulation stage is a compromise of two opposite factors.

### 3.2. Concentrating ^213^Bi Apart from ^225^Ac

The next point after separation of ^221^Fr from the mother column consists in selecting the conditions for concentrated ^213^Bi accumulation. ^221^Fr may be allowed to move long enough in a certain reservoir, for example, in form of tube in order to decay into ^213^Bi before the latter is captured [30]. Another approach is related to the retarding ^221^Fr in the appropriate chromatographic medium specific to ions of heavy alkali metals. A number of sorbents including AMP-PAN (Triskem Int.), Dowex 50 × 8 [30], and inorganic sorbent modified with nickel-potassium hexacyanoferrate(II) (sorbent T-35 manufactured by “Termoxid” company, Russia) [35] were tested. The present work focuses on the investigation of macroreticular cation exchange resin AG MP-50, which is also promising for ^213^Bi retention and consecutive extraction from the generator.

**Figure 5:**
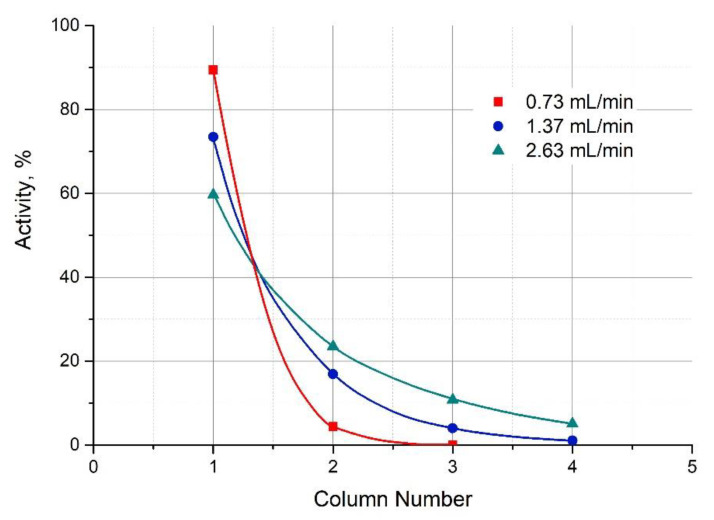
Distribution of ^213^Bi activity on the series-connected columns with 0.10 mL of AG MP-50, each depending on the flow rate of 0.25 М HNO_3_ eluent solution.

Adsorption of ^221^Fr and ^213^Bi onto AG MP-50 was studied according to the procedure described earlier in detail [30]. The mother ^225^Ac column was connected to four successive columns with AG MP-50 of a small volume (0.1 mL). The solution of 0.25 M HNO_3_ or 0.017 M NaCl was passed through the chromatographic system for no less than 4 h to attain transient equilibrium in the ^225^Ac → ^221^Fr → ^213^Bi → chain. Then the system was dismounted and γ-ray spectroscopic measurements of each column were performed. ^213^Bi distribution between four AG MP-50 columns as a function of flow rate of 0.25 M HNO_3_ solution is shown in Figure 5. Similarly, the ^213^Bi distribution for 0.017 M NaCl solution was obtained.

Following the procedure [30], the experimental data were used for estimation of capacity factors *k’* of Fr(I) onto AG MP-50 resin from 0.25 M HNO_3_ and 0.017 M NaCl solutions. Predictably, adsorption of ^213^Bi onto AG MP-50 was high and so it was only possible to set an upper limit for *k’* of Bi(III). The results are presented in Table 1 in comparison with the data reported earlier.

According to the results obtained, AG MP-50 can serve as a sorbent of second column for ^213^Bi accumulation. A small amount of AG MP-50 within 0.3–0.4 mL is sufficient for significant retention of ^213^Bi and this means that ^213^Bi can be further recovered from the generator in a more concentrated form.

**Figure 6:**
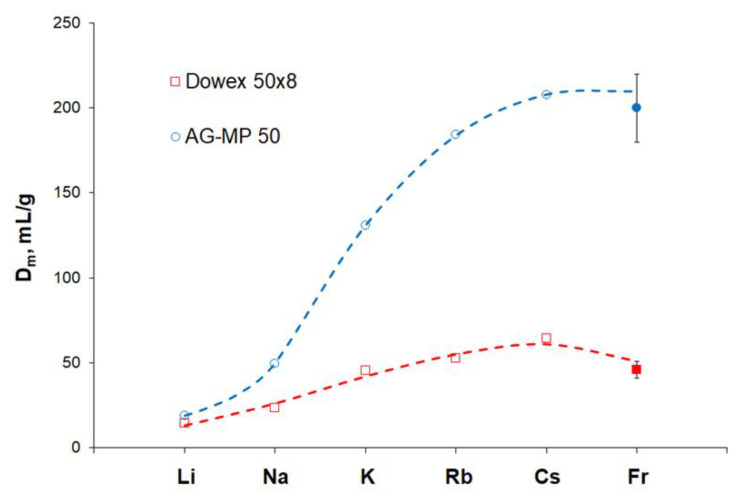
The mass distribution ratios *D_m_*, mg/mL, of alkali metal ions upon sorption onto Dowex 50 × 8 and AG MP-50 from 0.25 M nitric or hydrochloric acid. Literature data [28,38] are shown by empty circles and squares.

Comparison of ^221^Fr sorption on two cation exchange resins Dowex 50 × 8 and AG MP-50 from 0.25 M HNO_3_ solution reveals (Table 1) that the *k’* values of Fr(I) onto AG MP-50 is four times higher. Considering relatively equal chromatographic behavior of alkali metal ions in nitric and hydrochloric solutions [36,37,38], a similar tendency is observed for ions of other alkali metals (Figure 6).

Capacity factor *k’* was transformed into the mass distribution ratio D_m_ via the known relation: $D_{m}=k^{\prime }\frac{\varepsilon }{\rho_{app}}$, where ε denotes the fraction of sorbent free volume; $\rho_{app}$, denotes apparent density of sorbent. The manufacturer’s data for both Dowex and AG resin types are close and the values ε = 0.38 and $\rho_{app}$ = 0.8 g/mL may be used for a first approximation [39].

Considering that the alkali metal ions except Fr(I) were studied in batch experiments [28,38], the data consistency is quite satisfactory and allows us to conclude that the heavier the alkali metal ion, the more difference in its adsorption onto normal and macroreticular cation exchange resins is observed.

### 3.3. Extraction of ^213^Bi

The advantage of a two-column generator is the ability to perform desorption of the product with any of the most convenient complexing agents. In the case of a single-column generator, the eluent should be selected in such a manner as to efficiently transfer the daughter radionuclide into the solution and minimize the breakthrough of the parent radionuclide as well. If the parent and daughter radionuclides are kept in equilibrium and are simultaneously separated in space, for example, they are held by different columns, then there is no such limitation. Desorption of ^213^Bi can be carried out directly by a chelator or even by a chelator–protein conjugate, in which case the labeling takes place in the column and a ready-to-use radiopharmaceutical is obtained in the eluate. After desorption of ^213^Bi, the second column is washed with the solution used at the stage of its accumulation or it is replaced with a new one and the generator is ready for the next cycle.

**Figure 7:**
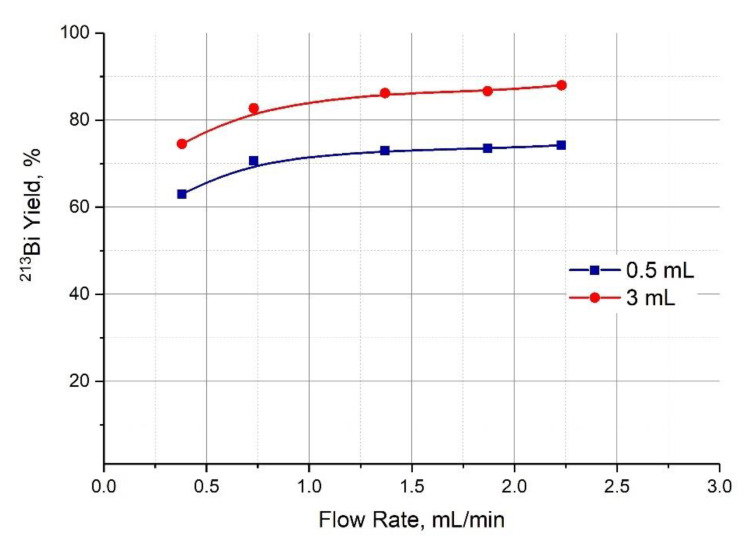
The elution efficiency of the generator “Afrabis” depending on the flow rate of the eluent (0.25 М HNO_3_) during circulation. The values for the first portion of eluate (0.5 mL) and for the whole fraction (3 mL) are given.

A 0.1M HCl/0.1M KI solution is used for ^213^Bi desorption in a single-column generator. This solution is convenient because Bi(III) forms strong complexes with halide ions, in particular, stable complexes ${\mathrm{BiI}}_{4}^{-}/{\mathrm{BiI}}_{5}^{2-}$ are formed with iodides [40], while Ac(III) does not form such strong complexes. The low acid content in the resulting eluate allows the quick creation of the medium suitable for labeling (pH 5–7) by adding a small volume of buffer. The desorption of ^213^Bi from a second column containing AG MP-50 resin with a 0.1M HCl/0.1M KI solution was studied. The overall efficiency of the "Afrabis" generator system was evaluated, provided that the accumulation stage was carried out by circulating 0.25 M HNO_3_ solution for ~4 h and ^213^Bi was stripped off with 0.1M HCl/0.1M KI solution. The efficiency of the generator “Afrabis” that depends on the flow rate of the eluent during circulation is shown in Figure 7. The efficiency values for the first portion of eluate (0.5 mL) and for the whole fraction (3 mL) are given. The desorption rate of the eluent was constant at 1 mL/min. It can be seen that the ^213^Bi yield increases with an increase of the flow rate during the accumulation stage and after 1 mL/min it reaches a plateau approaching 75% in the first 0.5 mL of the eluate and 90% in 3 mL.

A series of elutions with a constant flow rate at the accumulation stage and subsequent desorption of the product (1 mL/min) was performed to study the stability of the system. The average yield of ^213^Bi was 73 ± 2% in the first 0.5 mL of the eluate.

**Figure 8:**
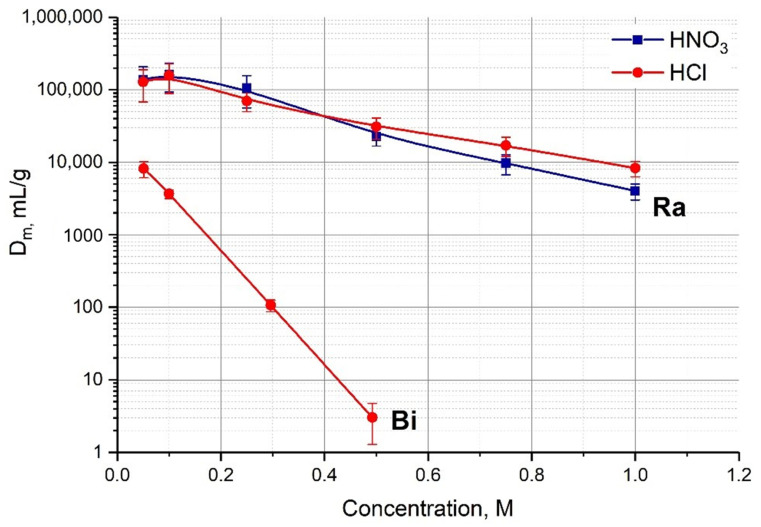
The mass distribution ratios D_m_, mg/mL, of Ra(II) and Bi(III) upon sorption onto AG MP-50 as a function of HCl or HNO_3_ concentrations.

The elution of ^213^Bi can also be carried out with 1 M HCl solution. The advantage of this approach is the high stability of a 1 M HCl solution unlike 0.1M HCl/0.1M KI and a small volume of eluent (0.5–1.0 mL) can be quickly evaporated using a special heater. The mass distribution ratios *D_m_*, mL/g, of Bi(III) upon sorption onto AG MP-50 as a function of HCl concentrations are given in Figure 8. It can be seen that D_m_ of Bi(III) becomes negligible for a HCl concentration higher than 0.5 M. At the same time, the retention of parent Ac in 1 M HCl is lower than in 0.1M HCl/0.1M KI, which can affect the service life of a single-column generator. In the case of a two-column generator, there is no such a problem and the efficiency for desorption with 1 M HCl is as high as for a 0.1M HCl/0.1M KI solution.

1,4,7,10-tetraazacyclododecane-1,4,7,10-tetraacetic acid (DOTA) and diethylenetriaminepentaacetic acid (DTPA) are the most often used in clinical medicine, although much of the new chelating ligands for Bi(III) are being synthesized and presently actively studied [41]. That is why these compounds were taken as test chelators for Bi desorption from the second column of the generator “Afrabis”.

**Figure 9:**
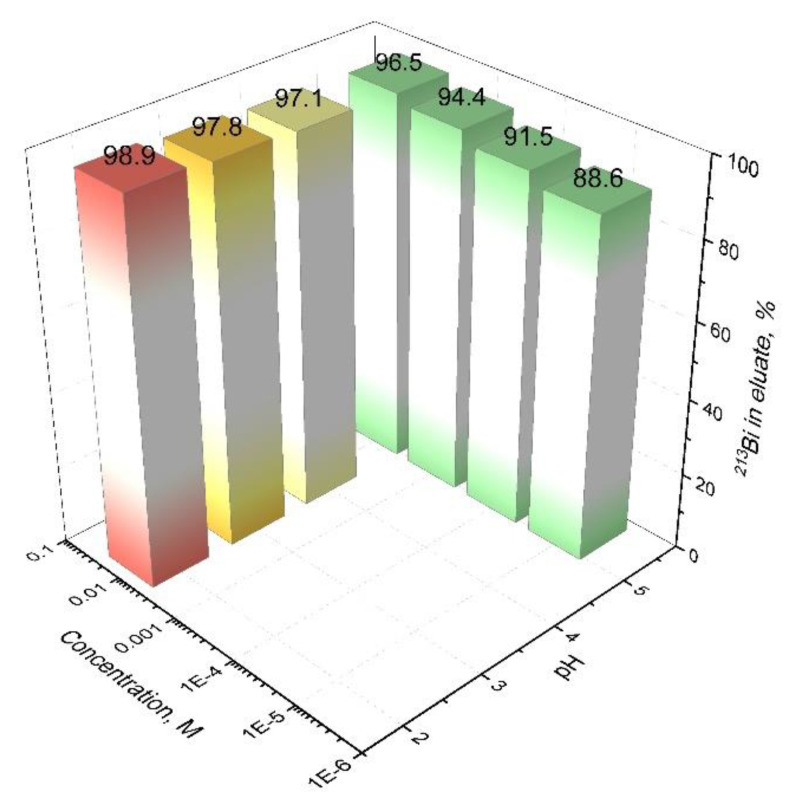
The elution efficiencies of Bi from the column with 0.4 mL AG MP-50 depending on the DTPA concentration and pH of the eluent. The volume of DTPA solution is 5 mL; flow rate—1.0 ± 0.1 mL/min.

The DTPA ligand forms a stable complex with Bi(III) (lgK (Bi-DTPA) = 35.2 [42]), which is formed in a few minutes and is already at room temperature. Due to its fast kinetics, DTPA is well suited for dynamic chelation, i.e., for desorption of ^213^Bi from the second column of the generator “Afrabis” in contrast to static chelation performed in batch conditions. The elution efficiencies of Bi from the column filled with AG MP-50 depending on the DTPA concentration and pH of the eluent are presented in Figure 9. Even at a concentration of 10^−5^ M, the desorption efficiency is close to 90% (^213^Bi distribution in other parts of the chromatographic system is not taken into account) and increases with an increase in the concentration of the ligand. The desorption efficiency also slightly increases with an increase of the pH value of solution. This effect is probably related to the kinetics of complex formation. At a constant DTPA concentration, the rate of complex formation decreases as pH rises. A similar dependence is observed for the kinetics of the formation of DTPA complexes with lanthanides [43].

**Figure 10:**
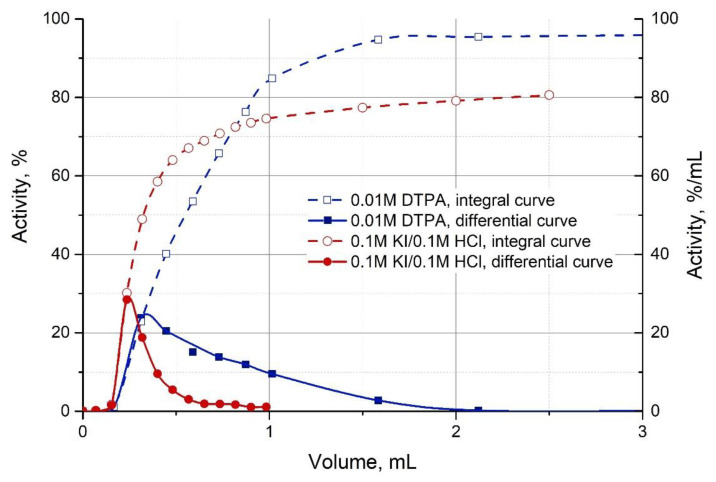
Integral (% of activity in the eluate, dashed lines) and differential (%/mL, solid lines) Bi elution curves with 0.1M HCl/0.1M KI and 0.01M DTPA (pH 5.3) from the column with 0.33–0.4 mL AG MP-50. Flow rate—1.0 ± 0.1 mL/min. ^213^Bi activity was corrected for decay.

Integral (% of activity in the eluate) and differential (%/0.1 mL) ^213^Bi elution curves are shown in Figure 10. The typical elution was conducted with 10^−5^ M DTPA with a pH 5.3. About 85% of ^213^Bi is eluted with the first 1.0 mL of the solution. For comparison, the same figure shows the elution curves of ^213^Bi with 0.1 M HCl/0.1 M HI for the conventional single-column generator based on AG MP-50 (discussed in 3.5). It can be observed that, in comparison with iodide complexes, the kinetics of Bi-DTPA complex formation is slower, but the overall desorption efficiency with DTPA solution is higher than with 0.1M HCl/0.1M KI.

Another widely used ligand is DOTA, an azacrown compound that is often referred to as the "gold standard" for many cations. It forms stable complexes (lgK (Bi-DOTA) = 30.3 [44]), while its main disadvantage is a slow rate of binding with most radionuclides. Thus, according to the literature data, the complexation with Bi must be carried out at heating and at 60–100 °C it will take 5–15 min [45]. For this reason, it is unsuitable to carry out the desorption of Bi(III) with a DOTA solution in a dynamic condition. However, the AG MP-50 sorbent can be quickly removed from the column and DOTA is labeled in a static regime by mixing the sorbent with the ligand solution at heating. It is found that the desorption with 3 mL 10^−5^ M DOTA solution (pH 5.3) under static conditions at a temperature of 90 ± 3 °С results in the binding of 80 ± 3% ^207^Bi in 5 min. In this case, there is no need to separate the non-complexed part of the radionuclide, since it remains bound on the cation-exchange sorbent.

### 3.4. Radionuclide Purity of ^213^Bi Extracted from the Generator “Afrabis” 

It is a matter for the near future when the part of ^225^Ac applied as a source for ^213^Bi generation is produced with the admixture of long-lived ^227^Ac. More stringent requirements will be imposed on the radionuclide purity of the ^213^Bi eluate in this case. In the generator “Afrabis”, due to the non-aggressive mobile phase (weakly acidic or neutral saline solution) used for the ^213^Bi accumulation stage, ^225,227^Ac radioisotopes are strongly bound onto the Actinide Resin column. Less than 0.2% of actinium was reported to be washed out of the mother column after one month of daily four hour generation cycles [46]. The actinium breakthrough was then effectively fixed on the second column preventing the final ^213^Bi from the impurity of actinium radioisotopes.

The influence of relatively long-lived decay products of ^227^Ac, namely ^227^Th and ^223^Ra, on the radionuclidic purity of ^213^Bi eluate is also important. Starting from the initial ^227^Ac/^225^Ac ratio ~0.2% at EOB and assuming null presence of ^227^Th and ^223^Ra at the moment of generator loading, the content of ^227^Ac, ^227^Th, and ^223^Ra grows up to 4.2%, 2.8%, and 1.7%, respectively, by the end-of-month generator operation. ^227^Th is retained by the sorbents of both columns no less firmly than actinium [33]. In contrast, the adsorption of ^223^Ra depends, to a greater extent, on solution acidity. For the example of 0.25 M HNO_3_ solution circulating in the system at the stage of ^213^Bi accumulation, the k’ value of Ra(II) on Actinide Resin falls within 20–30 [33], i.e., ^223^Ra is quite easily eluted from the mother column.

**Figure 11:**
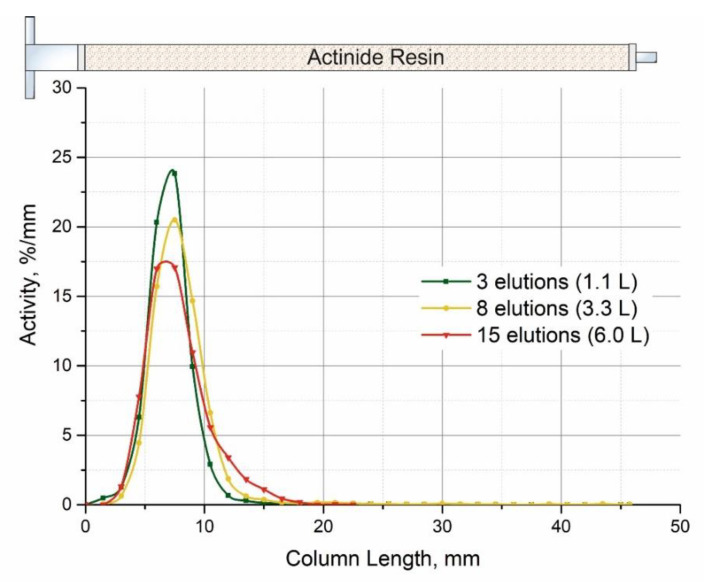
Distribution of activity along with the length of Actinide Resin column (0.4 mL) in the generator “Afrabis” depending on the number of elutions or the total volume of 0.25 M nitric passed through the column.

Scans of total activity produced by ^225,227^Ac and ^227^Th permanently adsorbed on the Actinide Resin column as a function of number of elutions or of a total volume of 0.25 M HNO_3_ solution passed through the column are shown in Figure 11. It can be observed that the initial location of ^225,227^Ac and ^227^Th in the first third of the column is slightly displaced after passing 6 L of the solution.

Since ^223^Ra is being removed in every generation cycle from the Actinide Resin column, the chromatographic system does not work as a double trap, which is the case of actinium and thorium radioisotopes. ^223^Ra is concentrated together with ^213^Bi on the second AG MP-50 column. According to the results presented in Figure 8, the D_m_ value of Ra(II) in 0.25 M HNO_3_ solution reaches 10^5^ mL/g, while the D_m_ Ra(II) in 0.1 M HCl/0.1 M HI solution used for ^213^Bi elution decreases down to 1.2∙10^4^ mL/g. Although the adsorption of radium on AG MP-50 is rather strong, some ^223^Ra breakthrough into the ^213^Bi eluate is possible, especially when ^213^Bi is eluted with certain ligand solutions such as DTPA.

**Figure 12:**
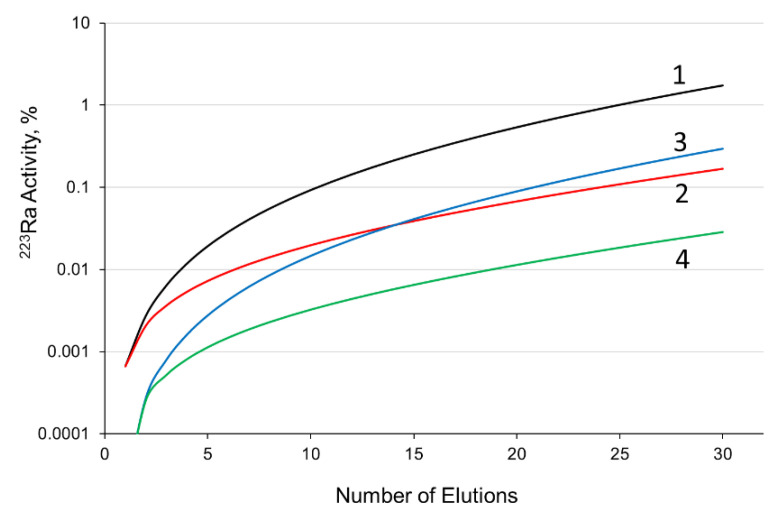
Estimation of maximal ^223^Ra amount in the generator “Afrabis” producing ^213^Bi once a day in a four hour cycle: 1—without preconditioning the mother column or replacing the second column (black line); 2—with replacing the second column after each cycle (red line); 3—with preconditioning the mother column before each cycle (blue line); 4—with preconditioning the mother column and replacing the second column every time (green line).

There were two opportunities considered for the periodic elimination of ^223^Ra from the generator. The first method was to replace the second AG MP-50 column with a new one after each generation cycle and, hence, ^223^Ra produced from ^227^Th between the cycles will be transferred to the second column. Otherwise, the mother column can be preconditioned before a generation cycle as it is shown in Figure 1, i.e., it can be preliminary washed with 0.25 M HNO_3_ or saline solution for removing ^223^Ra from the system. In this case, ^223^Ra produced from ^227^Th during only a single cycle is concentrated on the second column and accumulated from one cycle to another. Finally, we can combine the two opportunities. Figure 12 illustrates estimations of maximal ^223^Ra amount residing in the generator “Afrabis” in the course of a months work proceeding from the premises that ^213^Bi is generated once a day and one cycle’s duration is four hours.

Obviously, the least content of ^223^Ra is achieved by both preconditioning the mother column and replacing the second column. However, the generator maintenance becomes rather tedious so far and that is why the scheme including only the preconditioning was chosen for experimental trial. As a result of a months generator operation following the procedure given in Section 2.5, it was found that the impurities of ^225^Ac, ^227^Th, and ^223^Ra in 0.5 mL of the ^213^Bi eluate did not exceed 10^−6^% (detection limit), while the impurity of ^227^Ac estimated from the initial ^227^Ac/^225^Ac ratio was less than 10^−8^%.

### 3.5. Single-Column ^225^Ac/^213^Bi Generator with AG MP-50

Single-column "direct" ^225^Ac/^213^Bi generator with AG MP-50 resin [12] is the most common for ^213^Bi production. Clinical tests are carried out using such generators. The parent ^225^Ac is firmly retained by the sorbent and ^213^Bi is eluted with a complexing agent. The milking protocol was proposed at the Joint Research Centre in Karlsruhe (JRC, Germany). According to the protocol, ^213^Bi is eluted with 0.6 mL of 0.1 M NaI/0.1 M HCl solution from a column containing 0.33 mL of AG MP-50. The elution efficiency is reported to be 76 ± 3%, ^225^Ac breakthrough is less than 2·10^−5^%. The possible disadvantage of this generator is low radiation resistance of the organic sorbent [25]. Moreover, there are no data on the application of this generator with an accelerator-produced ^225^Ac containing an impurity of ^227^Ac and ^227^Ac decay products—^227^Th and ^223^Ra.

**Figure A1:**
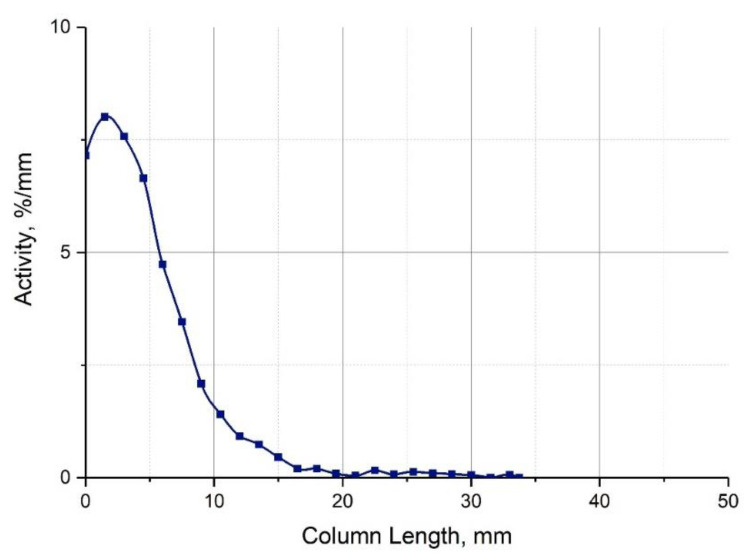
Initial distribution of ^225^Ac in the AG MP-50 column of ^225^Ac/^213^Bi generator.

A generator based on AG MP-50 was prepared in order to compare its performance with the generator system “Afrabis”. The generator was loaded, stored, and milked according to the procedure described [12] with the only difference being that ^225^Ac that is obtained by irradiation of thorium with protons (~0.2% ^227^Ac at the EOB) is applied. As a result of loading of ^225^Ac in a small volume of 4 M HNO_3_, the activity deposits on the top two-third part of the column. This approach was proposed by JRC to reduce the influence of radiation impact on the sorbent. Initial distribution of ^225^Ac in the AG MP-50 column of ^225^Ac/^213^Bi generator is shown in Figure A1. It can be seen that the distribution of activity is consistent with the literature data [12].

The results of the generator investigation carried out during 1 month are given in Table 2 in comparison with literature data and the generator “Afrabis” performance.

The typical integral and differential ^213^Bi elution curves with 0.1M HCl/0.1M KI from the single-column generator with AG MP-50 are shown in Figure 10. The elution efficiency of ^213^Bi for the single-column generator (67 ± 2% in 0.5 mL) is slightly lower than for the generator “Afrabis” (73 ± 2% in 0.5 mL). At the same time, the estimate of the breakthrough of the parent ^225^Ac (<3.5 × 10^−5^%) is consistent with the literature data, which was obtained for higher loaded ^225^Ac activity. The resulting ^225^Ac content limit for the generator system “Afrabis” is noticeably lower (<10^−6^%).

The difference in the degree of purification from ^223^Ra, which is a decay product of ^227^Ac, is significant; for a single-column generator, ^223^Ra activity concentration in the first portion of ^213^Bi eluate is 10^−4–^10^−3^%, while for the generator “Afrabis” the corresponding value is <10^−6^%. The obtained results are in accordance with theoretical views. Ac(III), Th(IV), and Ra(II) do not form strong iodide and chloride complexes, unlike Bi(III) [40]. Therefore, the retention of these ions by a strongly acidic cation exchange sorbent from the 0.1 M NaI/0.1 M HCl solution increases with their charges in the order Ra(II) < Ac(III) < Th(IV) [39].

It can be concluded that the developed generator “Afrabis” provides ^213^Bi with a higher radionuclide purity than compared to the conventional generator. The content of the parent radionuclide in ^213^Bi solution is at least one order of a magnitude lower and ^223^Ra is at least two orders of a magnitude lower. At the same time, the efficiencies of ^213^Bi extraction from both generators are similar.

## 4. Conclusions

A new two-column ^225^Ac/^213^Bi generator intended for using the parent ^225^Ac with ^227^Ac impurity is proposed and investigated. The formation and concentration of ^213^Bi on the second column is realized by continuous separation of intermediate ^221^Fr from ^225^Ac fixed on the first column. The generator is compact due to circulation of the mobile phase in a closed-loop circuit. In comparison with the generators described in literature, this provides a high product yield with a low breakthrough of actinium isotopes and ^227^Ac decay products. The concentration of ^213^Bi on a separate column allows us to desorb it with any convenient complexing agent, including chelation and labeling directly on the column.

## Figures and Tables

**Table 1:** Capacity factors k’ of Fr(I) and Bi(III) adsorbed onto various sorbents.

Stationary Phase (Sorbent)	Mobile Phase (Solution)	*k’* Fr(I)	*k’* Bi(III)
AMP-PAN (Triskem) ^1^	0.25 M HNO_3_	2.5·10^2^	>3·10^4^
Dowex 50 × 8 (Dow) ^1^	0.25 M HNO_3_	10^2^	>6·10^4^
T-35 (Termoxid) ^2^	1 M NH_4_Cl, pH 6.8	5·10^2^	>5·10^4^
AG MP-50 (BioRad)	0.25 M HNO_3_	4·10^2^	>5·10^4^
AG MP-50 (BioRad)	0.017 M NaCl, pH ~6	10^3^	>5·10^4^

Literature data: ^1^ [30], ^2^ [35].

**Table 2:** Results of ^213^Bi elution from the single-column ^225^Ac/^213^Bi generator with AG MP-50 resin.

^225^Ac/^213^Bi Generator	Efficiency of ^213^Bi Elution, %/in the First 0.5 mL of Eluate	Impurity in the First Eluate Portion of 0.5 mL, %			
		^225^Ac	^22^^7^Ac	^22^^7^Th	^223^Ra
“Afrabis”	73 ± 2	<10^−6^	<10^−8^ **	<10^−6^	<10^−6^
JRC (Karlsruhe, Germany)	67 ± 2 76 ± 3 [10] *	<3.5·10^−5^ 2·10^−5^ [10] *	<10^−6^ **	<10^−6^	10^−4^–10^−3^

* For first 0.6 mL of eluate; ** (estimated by ^225^Ac).

## Data Availability

Not applicable.
